# Network-based analysis of the sphingolipid metabolism in hypertension

**DOI:** 10.3389/fgene.2015.00084

**Published:** 2015-03-04

**Authors:** Mogens Fenger, Allan Linneberg, Jørgen Jeppesen

**Affiliations:** ^1^Department of Clinical Biochemistry, Copenhagen University HospitalHvidovre, Denmark; ^2^Research Centre for Prevention and HealthGlostrup, Denmark; ^3^Department of Cardiology, Glostrup University HospitalGlostrup, Denmark

**Keywords:** hypertension, sphingolipids, phosphatidate metabolism, redox metabolism, genetic networks, mutual information, epistasis, heritability

## Abstract

Common diseases like essential hypertension or diabetes mellitus are complex as they are polygenic in nature, such that each genetic variation only has a small influence on the disease. Genes operates in integrated networks providing the blue-print for all biological processes and conditional of the complex genotype determines the state and dynamics of any trait, which may be modified to various extent by non-genetic factors. Thus, diseases are heterogenous ensembles of conditions with a common endpoint. Numerous studies have been performed to define genes of importance for a trait or disease, but only a few genes with small effect have been identified. The major reasons for this modest progress is the unresolved heterogeneity of the regulation of blood pressure and the shortcomings of the prevailing monogenic approach to capture genetic effects in a polygenic condition. Here, a two-step procedure is presented in which physiological heterogeneity is disentangled and genetic effects are analyzed by variance decomposition of genetic interactions and by an information theoretical approach including 162 single nucleotide polymorphisms (SNP) in 84 genes in the sphingolipid metabolism and related networks in blood pressure regulation. As expected, almost no genetic main effects were detected. In contrast, two-gene interactions established the entire sphingolipid metabolic and related genetic network to be highly involved in the regulation of blood pressure. The pattern of interaction clearly revealed that epistasis does not necessarily reflects the topology of the metabolic pathways i.e., the flow of metabolites. Rather, the enzymes and proteins are integrated in complex cellular substructures where communication flows between the components of the networks, which may be composite in structure. The heritabilities for diastolic and systolic blood pressure were estimated to be 0.63 and 0.01, which may in fact be the maximum heritabilities of these traits. This procedure provide a platform for studying and capturing the genetic networks of any polygenic trait, condition, or disease.

## Introduction

Essential hypertension refers to hypertension with no known cause, affects approximately 30% of the adult population, and is a major risk factor for stroke, coronary incidences, and end-stage renal disease (Kearney et al., 2005). Several clinical and biochemical variables has been shown to be correlated to hypertension (Wang et al., 2007; Parikh et al., 2008; Singer and Setaro, 2008), but still the causes of essential hypertension remains elusive (Weder, 2007).

Blood pressure levels in a population constitutes an ensemble of polygenic conditions (Shih and O'Connor, 2008) meaning that the blood pressure is regulated by a plethora of integrated biochemical and physiological processes that are blue-printed in the genome (Fenger et al., 2011). Hypertension arise as a consequence of variations in these networks of intermingled processes increasing the propensity for developing hypertension conditional to a variable extent on non-genetic factors. Thus, blood pressure levels spans a scale of genetic information conditional on which non-genetic factors may influence the clinical outcome. However, the genetic causes of essential hypertension are largely unknown.

Several approaches including genome-wide association (GWA) studies (reviewed in Deng, 2007; Hamet and Seda, 2007; Shih and O'Connor, 2008) have extensively been used to define the genetics of blood pressure regulation and hypertension, but only a few potential genes have been associated with essential hypertension (Deng, 2007; Hamet and Seda, 2007; WTCCC, 2007; Ehret et al., 2008; Shih and O'Connor, 2008; Adeyemo et al., 2009; Levy et al., 2009; Newton-Cheh et al., 2009). In particular the large GWA studies have been somewhat disappointing as only sketchy information about the networks regulating the blood pressure have been provided. This may not be that surprising as the vast majority of analysis have been done as a search of monogenic effects in a case-control framework. Such approaches are at best simplifications of basic biological principles i.e., all biological processes are defined by interacting components (enzymes, metabolites etc…). For instance, epistasis (*in extensio* the interactions of the genes in the entire network) may be the most important genetic contribution to the variance of a trait, not the main effects (Fenger et al., 2008, 2011; Shao et al., 2008; Huang et al., 2012). Considering that the number of variations discovered runs in the millions, most networks (the sizes of which we do not know) will harbor thousands of variations in coding and non-coding, regulatory areas in principle defining as many networks as the number of combinations of variations. Some of these are not viable and hence never expressed, but still the number of networks are staggering (Fenger, 2012). This genetic heterogeneity is reflected in phenotypic heterogeneity, and hence a condition as hypertension is merely a clinical endpoint of diverse states of genetic networks and metabolic pathways in blood pressure regulation. Several approaches to include gene-gene interactions has been suggested including genetic algorithms and machine learning techniques (e.g., Routman and Cheverud, 1997; Culverhouse et al., 2002; Bureau et al., 2005; Zeng et al., 2005; Alvarez-Castro and Carlborg, 2007; Kang et al., 2008; Cantor et al., 2010; Wang et al., 2010), but the drawbacks are that specific genetic models are required, arbitrary data-reduction procedures are widely used, and many of the approaches requires that main effects are detected thereby excluding most genes from analysis.

**Figure 1:**
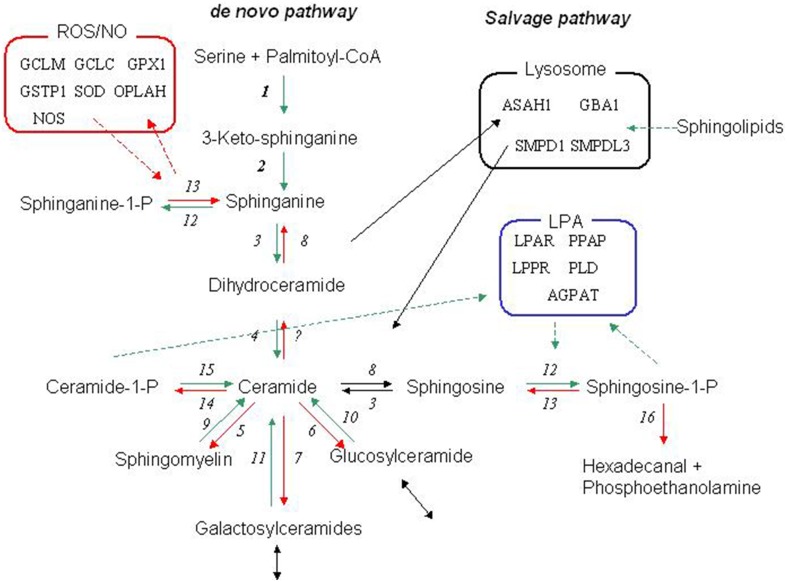
**The sphingolipid metabolic network**. The core biochemical network of the sphingolipid metabolism and the relation to the phosphatidate (LPA) and redox (Radical) networks. The canonical *de novo* pathway is initiated by a condensation of serine and palmitate by serine palmitoyltransferase (1). The product 3-keto-sphinganine is further processed to sphinganine by 3-ketodihydrosphingosine reductase (2), which can either by phosphorylated by sphingosine kinase (12) or converted to dihydroceramide by dihydroceramide synthase (3) and finally emerging as ceramide by the action of dihydroceramide desaturase (4). The faith of ceramide is then determined by the balance and interaction of several enzymes including ceramidase (8), sphingosine kinase (12), sphingomyelin synthase (5), sphingomyelinase (9), UDP-galactosyl ceramide glycosyltransferase (7)-galactosidase (11),UDP-glucose ceramide glycosyltransferase (6), and-glucosidase (10). Ceramide may also be phosphorylated to ceramide-1 phosphate by ceramide kinase (14), which may be reverted by a putative ceramide phosphatase (15). Sphingosine-1 phosphate may be dephosphorylated by sphingosine-1 phosphate phosphatase (13) or irreversibly degraded by sphingosine-1-phosphate lyase. Many of these enzymes has two or several isoforms. For a review see e.g., Lahiri and Futerman (2007). The salvage pathway (Lysosome) refers to the re-generation of sphingosine by acidic sphingomyelinase, galactosidase, and glucosidase that generates ceramides, which is further decomposed to sphingosine by acidic ceramidase. Please refer to Table S1a for abbreviation of the enzymes. For further information of the phosphatidate and redox metabolism related to the sphingolipid metabolism (see e.g., Ghelli et al., 2002; Won and Singh, 2006).

Previously we addressed the problem of resolving physiological heterogeneity of a population by implementing a latent class/structural equation modeling (LCA/SEM) framework using common physiological variables generally assumed to be related to cardiovascular conditions (Fenger et al., 2011). This approach revealed 14 distinct subpopulations with different propensity to develop hypertension embracing subpopulations with no hypertensive cases at all to subpopulations where the majority or all the subjects presented themself with hypertension. The significance of the genetic network of the sphingolipid metabolism in hypertension were evaluated by variance decomposition with focus on the *de novo* synthesis of sphingolipids and in particular the ceramide/sphingosine-1-phosphate rheostat (Fenger et al., 2011). The influence of sphingolipids on the vascular tone and hypertension is controversial as opposing vasodilatory and vasoconstrictive effects have been reported (Johns et al., 2001; Rosskopf et al., 2007; Alewijnse and Peters, 2008; Pavoine and Pecker, 2009; Feletou et al., 2011), particularly for the essential metabolites in the ceramide/sphingosine-1 phosphate (Cer/S1P) rheostat (Igarashi et al., 1999; Li et al., 2002; Ohmori et al., 2003; Hemmings, 2006; Alewijnse and Peters, 2008). In most tissues S1P has vasoconstrictive effects (Hemmings, 2006), but the regulation of vascular tone is vessel (organ)-dependent (Mulders et al., 2009; Fenger et al., 2011). Here, we extend our previous analysis (Fenger et al., 2011) including a comprehensive selection of genetic variations covering the sphingolipid metabolism and related processes (the redox and phosphatidate networks, Figure 1) and introduce an information theoretic analysis to assess the significance of genetic factors and their interaction. Even with the relatively small number of genetic variations included here the analytical procedures are highly involved, but most importantly, despite the genetic complexity of blood pressure regulation, almost all affected subjects could be captured by genotyping a few genes, although the predictive value of the compound genotypes varies considerably.

## Materials and methods

### Ethical statement

The MONICA study was conducted in accordance with the Second Helsinki Declaration and was approved by the ethics committee for Copenhagen County. Written informed consent was obtained from all participants, including permission to take part in genetic research.

### Model assumptions

**Figure 2:**
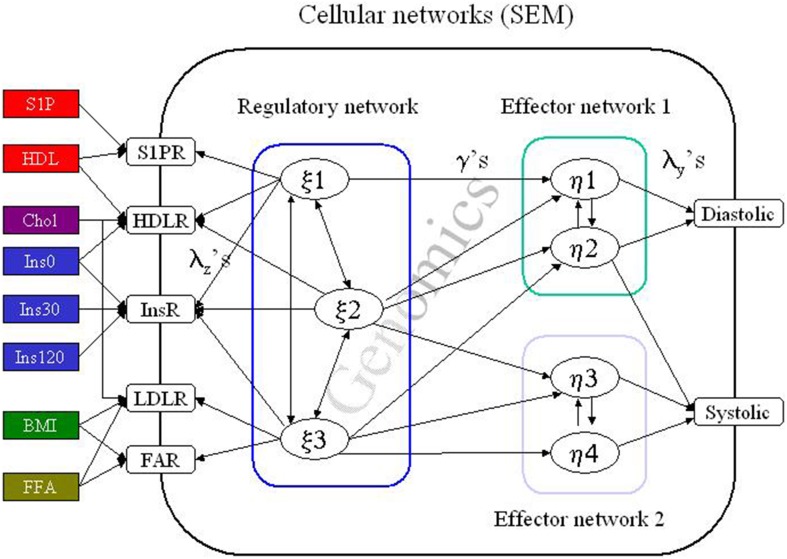
**The structural equation model (SEM)**. A simplified cartoon of the principal behind structural equation modeling (SEM). Here a cell is influenced by several “environmental” factors e.g., insulin (Ins) which induce a response in the cellular regulatory network cascade. The effect of this signal transduction is to induce or inhibit transcription of genes or genetic regulatory structures (Genomics). In addition, the signaling may directly influence the functionality of effector networks e.g., by promoting phosphorylation of effector proteins in all resulting in regulation of the blood pressure. In real life the pathway from the external factors to the ultimate response is much more complex as several cell types are involved in the local process (the vessel) and several tissues or organs will be involved (e.g., fatty acids generated in the liver) in a complex interactive network. The entire system is formulated by a complex set of matrix formulated regression algorithms, which may be proximal to the processes we actually can obtain information above or just by summary expressions. The subpopulations are then defined by simultaneous optimizing the SEM and the latent class (or rather latent profile) defining the population structure. A detailed account can be found in Muthen and Muthen (2004); Fenger (2008, 2012); Fenger et al. (2011).

The physiologically heterogeneity of diastolic and systolic blood pressures was resolved by partition of the study population by combined latent class analysis and structural equation modeling (LCA/SEM) into an ensemble of 14 physiological more homogeneous subpopulations, i.e., more accurate phenotypes were defined (Fenger et al., 2011). In this model it is assumed that the population consists of a mixture of subpopulations within which all variables in the model are only correlated through the latent SEM variables. No assumptions are made of the distribution of traits in the basic study population, but it is assumed that the traits are normal distributed in the subpopulations. Variables may not be exactly normally distributed in a tissue or an organism because of asynchrony of the dynamic processes in the cells (Muthen and Muthen, 2004), but the modeling approach used here is robust to minor deviations from normality. The latent SEM variables are composite variables as they include a plethora of processes that are not measured directly (Figure 2). All subjects are assumed to possess the same basic genetic structures, but vary in expression because of variability of any kind [single nucleotide polymorphisms (SNPs), deletions, insertions, copy number variation, non-coding RNA complexes, epigenetic diversity etc… ] in the genome. No genetic structure or model is assumed *a priori* except that all genes are part of functional genetic networks, which are embedded in the SEM model. The clinical endpoint, hypertension, is however not entered in the model, as the essential idea in the present approach is to model dynamic, biological processes using measured variables and to avoid more or less arbitrary defined clinical endpoint, which may not be entirely valid for all subjects or subpopulations, i.e., subjects are allocated to specific subpopulations based on objective measurements only.

The genome-wide genotype in a subject is fixed allocating an individual to a particular subpopulation together with subjects with similar physiological expressions. Implicitly, no subject transitions between subpopulations are possible. Only transitions from one level to another level of the blood pressure would be possible within the subpopulation, depending on the load of non-genetic factors and within limits defined by the subpopulation-specific genotype, i.e., the non-genetic factors operate conditional on a genetic framework that physiologically sets the range of a trait that cannot be exceeded. In the limit, each individual do of course defines its own subpopulation as no two individuals share the exact same genotype. However, the available physiological data have limited discriminatory power so the number of subpopulations is far less than the number of subjects included, and hence each subpopulation harbors subjects with similar, but distinct genotypes. Nevertheless, the partition of the population obtained serves its purpose to reduce variance within the subpopulations to such an extent that important parts of the genetic networks are captured.

The outcome of this approach is a classification of the subjects in the population in mutually exclusive subpopulations with distinct physiological metabolic states differing in propensity to evolve into hypertension. The best fitting model used blood pressure measurement in the supine position as the outcome (Fenger et al., 2011). The Mplus software used for the LCA/SEM analysis allocates a subject to a subpopulation for which it has the highest probability. However, allocation probabilities may occasionally be rather low reducing the robustness and power of further analysis.

For a detailed presentation and discussion of these complex issues, please see Fenger (2008, 2012); Fenger et al. (2011).

### Population

The study population is the Danish leg of the international MONICA-study (Jorgensen, 1987; Tunstall-Pedoe et al., 1999). In brief, random selected ethnic Danish women and men from the County of Copenhagen were enrolled in 1982 and re-invited for a reexamination in 1993–94 including 2556 subjects. Biochemical data were sampled including fasting glucose, insulin, lipids and cholesterol as well as anthropometric measures as height, weight, and waist and hip circumference. Blood pressure were measured by several modes including a standard “office” procedure in sitting and supine position. Please, see Fenger et al. (2011) for details.

### Genotyping

The 353 SNPs included in this study were extracted from NCBI database and included most of the exonic SNPs in the genes in the metabolic sphingolipid network as well as SNPs from the related redox and phosphatidate networks (Figure 1). Mostly missense and frameshift mutations in the exons in the selected genes was included in the genetic analysis. However, for genes in which no missense mutations has been reported synonymous mutations were genotyped to obtain maximum coverage of the genes. The details of the genes and SNPs are listed in Tables S1a,b. The genotyping was performed by KBioscience, Hoddesdon, Hertfordshire, United Kingdom. The call-rate was above 98.5%.

### Evaluation of genetic interactions

Epistasis was calculated by variance decomposition for all combinations of SNPs in all subpopulation as previously described (Fenger et al., 2011). Briefly, the two-gene/two-SNP variance of the systolic and diastolic blood pressure was stepwise decomposed calculating the effect size and the variance at each step for all combinations of SNPs in all subpopulations. SNPs not in Hardy-Weinberg equilibrium (HWE) were excluded from the analysis. Occasionally, SNPs in the entire population in HWE may be in disequilibrium in some subpopulations and visa versa. The evaluation of interactions were performed on the unfiltered subpopulations as well after applying restrictions of the minimal allele frequence of the SNPs and the probability of a subject to belong to his or hers designated subpopulation.

Two-gene interactions are actually measures of the mutual information (Brillinger, 2004; Hutter and Zaffalon, 2005) contributed to the network by the interactions. That is, if an interaction is detected it provides information about the network the amount of which depends on the effect size of the interaction. A weighted mutual information (WMI) score is therefore defined as the sum of the genetic effect of a composite genotype multiplied by its mutual information i.e.:

$$\text{WMI}=-\sum {\text{Geff}}_{\text{ij}}{\;}^{*}\;p_{\text{ij}}{\;}^{*}\text{ log}\left(p_{\text{ij}}/p_{\text{i}}{\;}^{*}\;p_{\text{j}}\right)$$

where Geff_ij_ is the effect size of the i-j genotype, *p*_ij_ is the frequence of the composite genotype, and *p*_x_ is the frequence of the individual SNP genotypes. In all, nine composite genotypes are evaluated for each two-SNP interactions. The mutual information [− ∑*p*_ij_^*^ log(*p*_ij_/*p*_i_^*^ *p*_j_)] is χ^2^-distributed with 4 df and only interactions significant after correction for multiple testing were included in the analysis.

### Case-control analysis

Comparisons of the distribution of genotypes between subpopulations (classes) were done in a classical case-control design in which each subpopulation were compared to each of the other subpopulations. Also, each subpopulation (“cases”) were compared to the remaining subjects (“controls”) in the study population. Significant two-SNP genotypes as evaluated by WMI (above) were analyzed in a case-control design in the entire population for diastolic and systolic hypertension separately.

### Statistics

Basic statistics were performed in SPSS v19.0 on a PC, or in LibreCalc on the Ubunto Linux platform. Bonferoni correction for multiple testing with a nominal *p*-value of 0.05 were applied to all analysis, unless stated otherwise. The Python packages Numpy, Scipy and Powerlaw were used in programmed algorithms. Plots were generated by QtiPlot v 0.9.8.8 on the Ubunto Linux platform.

A parallel, multi-threaded software package for variance decomposition, analyses of epistasis, mutual information analysis and topology of the networks is under development in Python and will be freely available shortly.

## Results

### General description of the population

Fourteen subpopulations were previously identified by latent class/structural equation modeling (Fenger et al., 2011) using blood pressure measurements as the outcome variables. Among several modes of blood pressure measurements the measurements in the supine position was used as this mode had the best goodness-of-fit of all modes in latent class/structural equation modeling. Subpopulation 1 (*N* = 39) was not further evaluated as none of the subjects were allocated to this subpopulation with a probability above 0.3. All the remaining subpopulations did contain subjects with an allocation probability of 0.9 or above although the number subjects and valid SNPs (exclusion of SNPs not in HWE) declined with increasing allocation probability (Tables S2, S3 for details for diastolic and systolic blood pressure, respectively). Thus, to obtain the maximal reliability the allocation probabilities of 0.9 or above was chosen for further analysis. Applying this restrictions and excluding subjects with missing values in LCA/SEM analysis reduced the study population to 722.

The 353 SNPs included were all annotated as polymorphic in NCBI dbSNP database, but only 160 turned out to be polymorphic in the MONICA population (Tables S1a,b). SNPs were only included in the analyses if the minor allele frequency (MAF) was above 0.01 to limit or avoid sporadic interactions, although the such excluded interactions may be real. Anyhow, cutting MAF at 0.01 or 0.05 only change the results marginally compared to not deleting any SNPs as he vast majority of MAFs were above 0.05. However, setting the MAF to 0.1 reduced the information gained from the genes and is therefore not appropriate.

The number of significant two-SNP interactions (epistasis) did not show a consistent pattern and differs between the subpopulations. In some subpopulations, including subjects allocated with a probability of 0.7 or above, the number of interactions was highest, but this it not the case in all subpopulations (Tables S2, S3). Restricting the MAF to above 0.01 reduced the numbers of epistatic interactions estimated by the variance decomposition, but the amount of interactions were still in the thousands.

The WMI was increased (in several cases substantially) when the allocation probability was increased for all MAF levels in all classes (Tables S2, S3). However, the number of interactions passing the WMI significant test (before and after correction for multiple testing) were drastically reduced (Tables 1, 2). The reason for these behaviors are two: the change in genotype distributions as subjects with less reliable class allocation were removed, and the change in effect size of the genotypes. Increasing the allocation probability essentially reduces “noise” in the sense of non-robust classification of subjects increasing the mutual information and hence the WMI. All interactions with significant mutual information are summarized in Tables S4, S5.

**Table 1:** **Summary of significant interactions of WMI in diastolic blood pressure**.

**Only SNPs with MAF of 0.01 or above is included**					
			**Epistasis**	**WMI Bonferoni corrected**	
**Subpopulation**	**Subjects**	**Valid SNPs**	**Number**	**Total WMI**	**Number**
2	44	140	8197	244	34
3	143	68	1734	3	9
4	16	121	5058	318	16
5	53	125	6360	281	39
6	82	137	7411	224	43
7	85	135	6774	264	34
8	12	110	516	173	4
9	83	130	6483	265	40
10	23	124	6987	318	18
11	90	126	5724	257	41
12	14	113	5914	470	14
13	61	123	5915	288	43
14	16	117	3818	414	9
	722		70,891	3521	344
	Fraction of possible epistasis		42.60%		
	WMI/epistasis ratio				0.49%
	Unique WMI-significant interactions			185	53.78%
Males	1258	100	2911	8.70	16
Females	1251	69	1539	2.28	6
	2509		4450	10.97	22
	WMI/epistasis ratio				0.49%

**Table 2:** **Summary of significant interactions of WMI in systolic blood pressure**.

**Only SNPs with MAF of 0.01 or above is included**					
			**Epistasis**	**WMI Bonferoni corrected**	
**Subpopulation**	**Subjects**	**Valid SNPs**	**Number**	**Total WMI**	**Number**
2	44	140	8195	405	32
3	143	68	1752	5	11
4	16	121	1057	428	13
5	53	125	6374	405	38
6	82	137	7413	356	39
7	85	135	6752	490	36
8	12	110	255	0	0
9	83	130	6530	389	37
10	23	124	6984	546	18
11	90	126	5686	403	32
12	14	113	5136	773	14
13	61	123	5927	485	40
14	16	117	3586	661	9
	722		65,647	5345	319
	Fraction of possible epistasis		39.45%		
	WMI/epistasis ratio				0.49%
	Unique WMI-significant interactions			182	57.05%
Males	1258	100	2958	11.67	8
Females	1251	69	1548	2.97	3
	2509		4506	14.64	11
	WMI/epistasis ratio				0.24%

Subpopulations 3, 8, and 11 differs from in this general analysis from the other subpopulations in several ways and will be treated separately below.

### Analysis of gender

Stratifying the population according to gender only provide little information about the networks. Although thousands of significant interactions were detected (59–66% of all possible) only a few accounted collectively for 50% or more of the WMI, which is anyhow low compared to the subpopulations extracted by the LCA/SEM procedure (Tables 1, 2). Four genes (GALC, FABP2, SMPD4, and ASAH2) accounted for this information.

The GALC (rs398607)/GALC(rs34362748) interaction was prominent in both gender and particular for diastolic blood pressure. The explained variance by this interaction in the gender partition was 8.7% or below. This SNP-combination was recovered in most but not all subpopulations although with very low information content (WMI). The prevalence of hypertension associated with this SNP-combination did not significantly differ from population prevalence of hypertension, however. The FABP2 (rs1511025)/FABP2 (rs1799883) interaction was recovered in some subpopulations, but all with very low WMI and none significantly influenced the prevalence of hypertension.

Interestingly, the two dominating interactions in the subpopulations (the SPHK1 and ASAH1 interactions, Tables 1, 2, and see below) were not detected, suggesting that, accepting the assumption that blood pressure states and hypertension are heterogeneous physiological conditions, simply partition of the population by gender was not sufficiently discriminatory. This is in accordance with LCA/SEM model implemented to define the subpopulations (Fenger et al., 2011), where gender did not enter as a co-variate at all. Thus, partition the population according to gender only reveals few significant interactions with marginal effects, which do not influence the prevalence of hypertension.

### Analysis of subpopulations

#### Variance decomposition

One of the most striking findings was that two interactions were present as the top-2 interactions in most subpopulations (Tables 1, 2): the SPHK1 (rs8176328)/SPHK1 (rs2247856) interaction were detected with the restrictions imposed (MAF of 0.01 and allocation probability 0.9) in all subpopulations except in subpopulation 3 for diastolic blood pressure and subpopulations 3 and 8 for systolic blood pressure. Similarly, the ASAH1 (rs1049874)/ASAH1 (rs1071645) interaction was detected as a top-2 interaction in all subpopulations except in the subpopulations 3, 8, and 11 for both diastolic and systolic blood pressure. This ASAH1 interaction was not detected in subpopulations 3 and 8 at all (Tables S4, S5). These two top interactions (termed *ubiquitous interactions*) were consistently detected regardless of MAF and allocation probability level except in subpopulations 3, 8, and 11 (Tables S2, S3).

In subpopulation 8 the interactions determined by variance decomposition were increasing as the allocation probability was raised to 0.7, but was drastically reduced when allocation probability threshold was set at 0.9 and above with a concomitant reduction in number of subjects in this class from 88 to 12. The number of interactions were reduced considerably to approximately 7.8 and 3.8% (diastolic and systolic blood pressure, respectively) of the maximum amount of interaction encountered at a probability allocation threshold of 0.7 (Tables S2, S3).

Regression of the relative genotype/phenotype variance on the phenotypic variance for the two-SNP genotypes almost perfectly fit a straight line (*R*^2^ ~ 0.98), that is the larger the genetic influence of the two-SNP genotypes the lower phenotypic variance and hence a lower impact of non-genetic factors, supporting the assumption that the genetic network sets the level and the range of variation possible for an individual as well in a homogenous subpopulation. It could be expected that the relative epistasis was correlated to the genotypic variance too, but the correlation turned out to be were low (not shown). The two two-SNP genotypes with the highest genotype/phenotype variance (and lowest phenotypic variance) are the SPHK1 rs8176328/rs2247856 genotypes (distance 12 nt) and the ASAH1 rs1049874/rs1071645 genotypes (distance 1482 nt). This is consistently seen for all subpopulations except subpopulation 3 and partly subpopulations 8 and 11 (Tables S4, S5).

#### Weighted mutual information, WMI

In all, only approximately 0.5% of all interactions detected by variance decomposition was recovered after filtering for significant mutual information (Tables 1, 2). A total of 344 significant WMIs were detected for diastolic blood pressure and 319 for systolic blood pressure (Tables S4, S5, respectively) distributed over the subpopulations. Of these 663 WMIs 182 represents unique two-SNP interactions in the entire ensemble of subpopulations for both traits, while additional three were detected for diastolic blood pressure only, amounting to more than 50% of all WMI-significant interactions for the two blood pressure measurements separately (Tables 1, 2, and Table S8). This is almost 4-fold more interactions compared to partition of the study population only by gender (not shown).

The total WMI were at similar levels for the subpopulations except for subpopulation 3 and 8 using the restrictions imposed (see below). Slightly more than one third of the two-SNP WMIs (39.2% for diastolic blood pressure and 35.5% for systolic blood pressure) were private in the sense that they only occurred in one subpopulation (Tables S4, S5). Most of these are common to both traits but have very low WMI. A few interactions did however have a substantial WMI particularly in subpopulations 4, 8, 12, and 14, and thus may have a significant role in defining the subpopulations (Tables S4, S5).

Subpopulation 3 showed a different pattern of interactions as the ubiquitous interactions were not captured. The number of valid SNPs i.e., non-monomorph SNPs which are in HWE was reduced significantly to almost half compared to the other subpopulations. The vast majority of SNPs were excluded because they “turned” monomorph and hence non-informative, which particularly included the SNPs defining the ubiquitous genotypes except the SPHK1 rs8176328. ASAH1 was not detected at all. Consequently the major source of information was missing and the information retained by the remaining SNPs was extremely low amounting to 1% or less compared to the other subpopulations (Tables 1, 2). The low WMI and genetic variance precluded this subpopulation from further analysis as any inferences would be highly unreliable.

Three ASAH1/ASAH1 interactions were detected by the WMI analysis in subpopulation 8 but not the ubiquitous combination mentioned above and they were not significant even at the nominal level of 0.05 (not shown). In contrast, the ubiquitous SPHK1/SPHK1 interaction was detected for diastolic blood pressure but not for systolic blood pressure though (Tables S4, S5). However, relaxing the strict allocation probability threshold from 0.9 to 0.7 the two ubiquitous interactions were detected with high probability in both diastolic and systolic blood pressure in this subpopulation and the general pattern of interactions was similar to the other subpopulations. Thus, the general allocation probability thresholds was somewhat arbitrarily set at 0.9, but a few lower allocation probability thresholds should be tested to avoid serious loss of information, as in this case could lead to the erroneous conclusion that the sphingolipid metabolism do not influence the blood pressure regulation in subpopulation 8, while in fact the contrary is the truth.

The ubiquitous SPHK1/SPHK1 interaction was highly significant for diastolic and systolic blood pressure and possessed high WMI values in subpopulation 11. In contrast, although ASAH1/ASAH1 interactions were detected by the WMI analysis, the ubiquitous combination above were not detected. The ASAH1/ASAH1 interactions was however “substituted” by a SMPD4 (rs76033185/ rs79875317; 20,267 nucleotides apart) with high WMI. SMPD4 codes for the neutral membrane sphingomyelinase 3 generating ceramide, while ASAH1 codes acid ceramidase generating sphingosine in the salvage pathway (Figure 1). Thus, at least theoretically, the ceramide/sphingosine-1 phosphate rheostat in subpopulation 11 is balanced in favor of ceramides, while the opposite is the case of all the other subpopulations except subpopulation 3. Hence, at least two basically different metabolic pathways in the sphingolipid metabolism is associated with blood pressure regulation.

#### Trait comparisons

Comparisons of trait difference conditional on two-SNP genotypes are shown in Tables S6, S7 for diastolic and systolic blood pressure, respectively. At the nominal significance level 336 and 304 two-SNP genotype combinations differed in diastolic and systolic mean values, respectively. However, only two comparisons remained significant after correction for multiple testing for diastolic blood pressure. Both were combinations of SNPs in SPHKAP, sphingosine kinase 1 interacting or anchoring protein. They were detected in just short of 50% of the diastolic hypertensive subjects. In the case of systolic blood pressure four SNPs in four genes were significant, but the prevalence in systolic hypertensive subjects were rather low. Nevertheless, one combination, the GALC rs398607 CC genotype combined with the CERK rs36211083 TT genotype, was associated with increased systolic blood pressure (see also Table 3) and may be clinically useful.

**Table 3:** **Prevalence of affected for two-SNP genotypes**.

							Fraction (%)^*^	
**SNP1**	**Gene1**	**Genotype1**	**SNP2**	**Gene2**	**Genotype2**	***p*-value**	**Genotype**	**Population**
**DIASTOLIC BLOOD PRESSURE**								
**Genotypes above the threshold**								
rs865832	SGPL1	TT	rs12770335	SGPL1	GG	0.0138	50	1.62
rs309087	LPPR5	CC	rs5186	AGTR1	CC	0.0279	50	1.3
rs3734462	AGPAT4	TT	rs1799983	NOS3	TT	0.0279	50	1.3
rs1799883	FABP2	AA	rs1799983	NOS3	TT	0.0220	45.45	1.62
rs28385609	SMPDL3A	TT	rs2003149	KDSR	*GA*	0.0182	36.36	2.6
rs309087	LPPR5	CC	rs5186	AGTR1	CA	0.0401	34.78	2.6
rs243887	SPTLC3	TT	rs1071645	ASAH1	**AG**	0.0123	26.97	7.79
rs243887	SPTLC3	TT	rs1049874	ASAH1	GA	0.0130	26.67	7.79
rs41292584	SMPDL3A	*CT*	rs1799983	NOS3	GG	0.0308	25	7.47
rs41292584	SMPDL3A	*CT*	rs3739709	LPAR1	CC	0.0313	23.53	10.39
rs1138439	PPAP2C	TT	rs12195587	ELOVL2	*TC*	0.0451	23.39	9.42
rs1138439	PPAP2C	TT	rs2003149	KDSR	*GA*	0.0377	23.13	11.04
rs3811514	SPHKAP	GG	rs3828161	SPHKAP	AA	0.0371	22.17	14.61
**Genotypes below the threshold**								
rs1799983	NOS3	**GT**	rs2566514	NOS3	CC	0.0008	10.18	16.23
rs3739968	ASAH2B	GG	rs1071645	ASAH1	GG	0.0341	10.16	6.17
rs285	LPL	TT	rs320	LPL	GT	0.0224	10.14	7.14
rs3739968	ASAH2B	GG	rs1049874	ASAH1	AA	0.0314	9.94	5.84
rs1799983	NOS3	**GT**	rs3828161	SPHKAP	**GA**	0.0123	9.87	7.47
rs1130233	AKT1	*AG*	rs3828161	SPHKAP	**GA**	0.0215	9.84	6.17
rs6109692	SPTLC3	*CT*	rs2241883	FABP1	**GA**	0.0430	9.79	4.55
rs11657217	ENPP7	GG	rs3734462	AGPAT4	*TC*	0.0479	6.67	1.3
rs6511701	S1PR5	*GT*	rs1695	GSTP1	GG	0.0358	6.15	1.3
rs11657217	ENPP7	GG	rs3170633	GCLM	GG	0.0010	4.35	1.3
rs4880	SOD2	TT	rs11657217	ENPP7	GG	0.0253	4.17	0.65
rs5186	AGTR1	CC	rs67319648	PPAPDC1A	*CT*	0.0485	3.23	0.32
**SYSTOLIC BLOOD PRESSURE**								
**Genotypes above the threshold**								
rs398607	GALC	CC	rs36211083	CERK	TT	0.0130	100	0.67
rs398607	GALC	TT	rs1805078	GALC	**TC**	0.0124	80	0.89
rs1130233	AKT1	AA	rs3828161	SPHKAP	GG	0.0302	66.67	0.89
rs243887	SPTLC3	TT	rs2241883	FABP1	GG	0.0206	50	2.01
rs1799983	NOS3	**GT**	rs3828161	SPHKAP	GG	0.0236	42.86	2.68
rs1130435	FABP6	CC	rs7157599	DEGS2	GG	0.0296	38.64	3.8
rs7850023	MIR4668	*AG*	rs5186	AGTR1	CC	0.0400	36.73	4.03
rs5186	AGTR1	AA	rs2301022	GCLM	AA	0.0169	33.63	8.5
rs1476387	SMPD2	TT	rs3739709	LPAR1	*CT*	0.0312	33.33	7.61
rs3739968	ASAH2B	**GA**	rs4918	AHSG	GG	0.0178	33.07	9.4
rs285	LPL	CC	rs1695	GSTP1	AA	0.0136	31.43	14.77
rs12195587	ELOVL2	*TC*	rs1476387	SMPD2	GG	0.0183	30.84	14.77
rs1138439	PPAP2C	TT	rs7302981	CERS5	CC	0.0408	30.77	10.74
rs6511701	S1PR5	*GT*	rs1695	GSTP1	AA	0.0123	30.63	18.57
rs285	LPL	CC	rs7157599	DEGS2	AA	0.0321	29.8	16.33
rs12195587	ELOVL2	*TC*	rs8176328	SPHK1	AG	0.0182	29.77	20.58
rs12195587	ELOVL2	*TC*	rs2247856	SPHK1	TC	0.0195	29.65	21.03
rs12195587	ELOVL2	*TC*	rs698912	COL4A3BP	AA	0.0407	28.42	23.71
**Genotypes below the threshold**								
rs6511701	S1PR5	GG	rs1868158	SMPD3	*AG*	0.0492	19.35	23.94
rs12195587	ELOVL2	CC	rs2247856	SPHK1	TC	0.0338	19.21	26.17
rs11657217	ENPP7	CC	rs402348	KDSR	CA	0.0359	17.34	9.62
rs285	LPL	TT	rs328	LPL	CC	0.0183	17.32	11.86
rs1799983	NOS3	GG	rs3828161	SPHKAP	**GA**	0.0119	16.33	8.95
rs3811515	SPHKAP	**GT**	rs16824283	SPHKAP	**CG**	0.0137	16.32	8.72
rs1138439	PPAP2C	CC	rs36211083	CERK	*TC*	0.0488	15.18	3.8
rs398607	GALC	TT	rs36211083	CERK	*TC*	0.0191	13.13	2.91
rs1049874	ASAH1	AA	rs4808863	CERS1	TT	0.0237	12.16	2.01
rs1071645	ASAH1	GG	rs4808863	CERS1	TT	0.0237	12.16	2.01
rs243887	SPTLC3	TT	rs1049874	ASAH1	AA	0.0268	9.8	1.12
rs243887	SPTLC3	TT	rs1071645	ASAH1	GG	0.0192	9.43	1.12
rs320	LPL	GG	rs328	LPL	CC	0.0282	9.09	0.89
**COMMON TO BOTH DIASTOLIC AND SYSTOLIC BLOOD PRESSURE**								
**Genotypes above the respective thresholds**						**Diastolic/systolic blood pressure**		
rs79875317	SMPD4	AA	rs1695	GSTP1	GG	0.002/0.001	71.43/54.17	1.62/2.91
rs76033185	SMPD4	GG	rs1695	GSTP1	GG	0.008/0.003	66.67/83.33	1.30/1.12
rs1138439	PPAP2C	TT	rs36211083	CERK	TT	0.044/0.038	44.44/55.56	1.30/1.12
rs2297568	SPTLC1	AG	rs3828161	SPHKAP	GG	0.010/0.001	37.50/54.17	2.92/2.91
rs12888666	GALC	AA	rs1695	GSTP1	AA	0.049/0.013	25.00/35.71	6.82/6.71
rs1130233	AKT1	AA	rs243887	SPTLC3	TT	0.014/0.015	50.00/60.00	1.62/134
rs11657217	ENPP7	*CG*	rs402348	KDSR	CA	0.016/0.043	22.27/29.3	18.51/16.78
**Genotypes below the respective thresholds**								
rs3734462	AGPAT4	*TC*	rs1049874	ASAH1	AA	0.020/0.043	9.73/16.76	5.84/6.94
rs11657217	ENPP7	GG	rs402348	KDSR	CA	0.013/0.043	4.76/29.30	0.97/16.78

Prevalence of diastolic hypertension in the population 16.13% (threshold).

Number of subjects with diastolic hypertension captured by the significant genotypes 253 (82.14% of all affected).

Prevalence of systolic hypertension in the population 23.40% (threshold).

Number of subjects with systolic hypertension captured by the significant genotypes 436 (97.54% of all affected).

Genotypes in bold, missense mutations; genotypes in italic, synonymous mutations.

For gene abreviations, please see Supplemental Data Table S1a.

* Freqencies of the affected by the two-SNP genotype (Genotype) and frequencies of the two-SNP genotype in the population (Population).

#### Heritabilities

**Figure 3:**
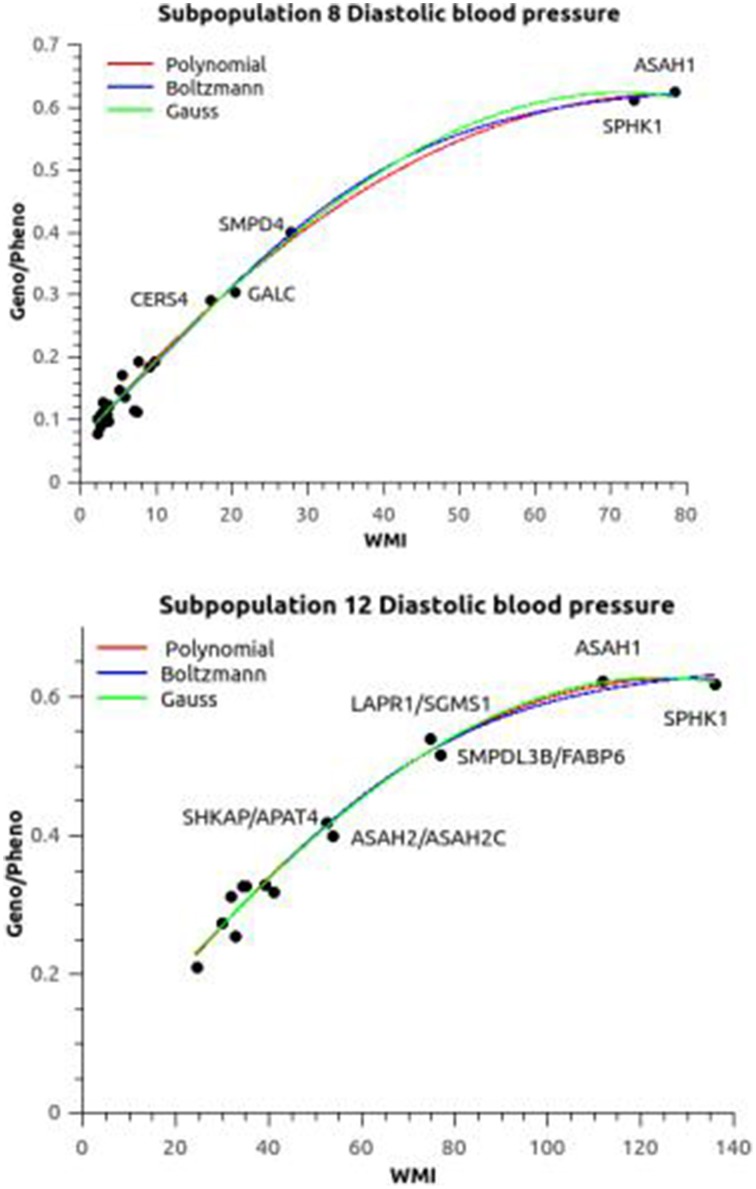
**Association between relative epistasis and WMI**. The plots shows the heritability (Geno/Pheno) of the diastolic blood pressure as a function of the weighted mutual information (the plots of systolic blood pressure are similar). Subpopulation 8 was evaluated at an allocation threshold of 0.7 in contrast to 0.9 for all the other subpopulation (see Text for discussion). The three fitted plots [polynomial, Boltzmann (sigmoid), and Gaussian] were almost overlapping. Apart from the ubiquitous ASAH1 and SPHK1 haplotypes there is a difference in the ranking of the next interactions between the subpopulations. Similar differences in patterns were seen for the other subopulations.

The heritability of blood pressure regulation as a function of the WMI is plotted in Figure 3. The three curve fitting methods polynomial, Boltzmann (sigmoid), and Gaussian gave almost the same heritabilities for blood pressure regulation except for subpopulation 3. The broad sense (all genetic variance included) heritabilities *h*^2^ = genotypic variance/phenotypic variance were estimated to be 0.626/0.012 for both diastolic and systolic blood pressure. The Boltzmann fit gave slightly higher values (0.70 and 0.69 for diastolic and systolic blood pressure, respectively). Subpopulation 8 was included in these calculation using a allocation probability threshold of 0.7 rather than 0.9, but still the curve fittings converged in a few iterations for the Boltzman and Gauss fits. The only difference was that the correlation between heritability and phenotypic variance showed a exponential decay (*R*^2^ ~ 0.98) in contrast to the remaining subpopulations, where the correlation was linear (*R*^2^ ~ 0.98). In contrast, subpopulation 3 did not fit into this general pattern. The heritability implied by the sphingolipid metabolism was only ~0.07, but still significant.

The two ubiquitous interactions defined by ASAH1 and SPHK1 were supplying the most information in all subpopulations except in subpopulation 3. These two genes are pivotal in the ceramide/sphingosine-1 phosphate rheostat: ASAH1 codes for acid ceramidase generating sphingosine and SPHK1 phosphorylate sphingosine to generate sphingosine-1 phosphate (Figure 1). The remaining interactions had various impact on WMI, the most notable differences illustrated by comparing subpopulation 8 and 12 (Figure 3). SMPD4, coding a neutral sphingomyelinase operating at the plasma membrane generating ceramide is prominent in subpopulation 8, while the acid sphingomyelinase (SMPDL3B) operating in the salvage pathway, i.e., in the lysosomes, did carry major information of the sphingolipid network in subpopulation 12. Also, the redox network (represented by GALC) seems to be of major importance in subpopulation 8.

Interactions/heritabilities not annotated in Figure 3 can be found in Tables S4, S5.

#### Case-control analysis

Assessment of differences in single-SNP or single-gene genotype distributions between subpopulations or individual subpopulations and the rest of the study population resulted in detection of some significant SNPs. However, less than 5% of the valid SNPs appeared to be potential discriminatory, no consistent pattern of SNPs were detected, and the *p*-values were mostly marginal, and no significant differences remained after correction for multiple testing.

In contrast, when two-SNP combinations were analyzed in the case-control design, 1755 and 1728 two-SNP combinations were detected for diastolic and systolic blood pressure, respectively, when WMI-significant interactions were exclusively included. Of these 34 and 40 were significant associated with the prevalence diastolic and systolic hypertension, respectively (Table 3). Approximately two-thirds were significantly associated with increased prevalence of diastolic or systolic hypertension applying the general accepted cut-off of 90 mmHg and 140 mmHg for diastolic and systolic blood pressure, respectively. The remaining interactions were associated with decreased prevalence. Most, but not all interacting SNPs turned out to be homozygous, suggesting the traditional concept of recessive genes, i.e., that genes (or rather genetic variations) has to be homozygous to affect a trait. The vast majority of two-SNP interactions were specific to diastolic or systolic blood pressure, respectively (Table 3). Only 9 of the 65 (unique) interactions (13.8%) were common to both blood pressure measurements.

### Network interactions

The interaction pattern is very complex even with the relative few two-SNP/gene interactions having an impact on the prevalence of hypertension (Table 3). The complexity extends to the metabolic pathways, but we only have a rudimentary knowledge of the regulation of these networks and pathways. In addition, there were striking difference in interaction patterns between diastolic and systolic blood pressure (Table 3). A discussion of the interactions is therefore highly tentative so only a few interactions are evaluated (see Table S1).

The two SNP-interactions, SPHK1 and ASAH1, are fixed at two haplotypes or alleles as only two homozygotes and one composite heterozygote were detected out of nine theoretically possible genotypes. The distance between the two ASAH1 SNPs is 1484 bp, while it is only 12 bp between the two SPHK1 SNPs (see however Table S2). These genotypes represented by far the largest amount of information (WMI) but did not influence the prevalence of hypertension (Table 3). In addition, there was no significant difference in mean values between the three genotypes for each of the genes and could not *per se* set different levels of blood pressure. However, as described above, the higher the WMI the larger amount of phenotypic variance is determined by the genotypes, i.e., the more the trait is fixed in the genome. SPHK1 and ASAH1 presented themself with the highest WMI, which can be interpreted as these SNPs or genes provides the most information of the genetic influence of the trait variation, but do not necessary set the actual level of the blood pressure. The epistasis defined by these two genes are intragenic defining three basic haplotypes each.

#### Topology

A plethora of network descriptors are available, but here we limit the discussion to the node degree distribution and the much applied power law and Poisson distributions of the WMI data sets. The power law is here defined as p(x)~x^−α^ and has obtained quite an interest as many data-structures seems to follow this simple distribution with the α-value generally estmated to be in the interval (2,3). Most of the classes do have α-values in this range, with a few classes outside this range for the nominal significant WMI (Table S9). However, only approximately 8–59% of the WMI values were captured by the power law or by any other heavy-tailed distributions tested (lognormal, exponential). The latter was improved considerably after correction for multiple testing, but apart from the lower α-values encountered, the variance of the α-values increased substantially. The nominal WMI data points not included in the power law distribution did not fit any of several other tested distributions (normal, lognormal, exponential, uniform, Poisson, and several more). However, in WMI data set corrected for multiple testing the remaining data after extractionof the power law data did fit Poisson distributions.

The degree sequences of the SNPs and genes for the WMI data sets show a similar pattern of distributions as for the WMI data sets themselves. The gene degree sequences for diastolic and systolic blood pressure are almost identical (Table S10). Several genes in the sphingolipid metabolic network seems to qualify as hubs including sphingosine kinase 1 (SPHK1), its regulatory protein SPHKAP, ceramide synthase 4 and galactocerebrosidase. Acid ceramidase (ASAH1) appears as a hub in the salvage pathway, and this enzyme together with SPHK1 are the two metabolic “endpoints” in the regulation of the blood pressure (see above and Figure 2). The source of fatty acids for synthesis of sphingolipids seems to be derived from circulating triglycerides suggested by the hub-like status of lipoprotein lipase (LPL) and fatty acid binding protein 2 (FABP2) (Table S10). Finally, the ROS and NO metabolisms are of central importance in the blood pressure regulation (Igarashi and Michel, 2009; Omo et al., 2014), which is supported by the high connectivity of the genes including links to genes in the other networks. In contrast, the LPA network seems only to be modestly connected, but this could be caused by the low amounts of selected SNPs that turned out to homozygous or were dismissed during analysis.

It should be kept in mind that the above conclusions are for the most part conjectural founded on the “naive” assumption that a higher degree of a gene equals importance. The real importance of a gene is the information it supplies by interaction with the other genes. Nevertheless, the above analysis is supportive of the conclusions obtained from the WMI analysis.

## Discussion

All populations are heterogenous due to the vast genetic variation present, and all genes only operates in integrated networks (Fenger, 2014). A recent (and striking) example of the importance of the genetic architecture in host-environment interactions has emerged, in which the host genotypes determines the environments influence, here the Ebola virus, on phenotypic outcome is firmly established (Rasmussen et al., 2014). These fundamentals are supported here: at least 14 subpopulations have been defined previously only including physiological variables related to blood pressure regulation in the partition analysis (LCA/SEM); and the blood pressure is governed by several metabolic networks that are integrated by complex communication pathways within and between the networks. Three metabolic networks were the primary targets for the analysis, but it is clear that the complexity extends beyond these three networks including communication with extracellular sources. The ceramide/sphingosine-1 phosphate rheostat is a well-established, delicate balanced communication system that roughly speaking has opposite effects and was shown to be pivotal in most subpopulations. Many more balanced feed-back systems are present however, which are corroborated by the genetic analysis present here.

Detection of genes and extra-genic variations of importance relies heavily on the reliability and accuracy of the phenotypes. This is clearly seen by comparison of the gender-based partition with the physiologically modeled partition, where there was more than a 300-fold increase in genetic information provided by the interactions, and more than a 15-fold increase in the number of significant interactions when corrected for multiple testing (Tables 1, 2). Most intriguingly, none of the few interactions detected in the gender partition analysis was recovered under more stringent conditions as influencing the prevalence of hypertension (Table 3). This could (cautiously) be interpreted as false positive results due to the coarse-grained partition of the population imposed by gender, but more importantly the partition of the population into more homogenous subpopulations using physiological variables in an latent class/structural equation modeling (other partition approaches are possible) is a prerequisite of obtaining information about the genetics of a trait or condition to any substantial and interesting extent.

The WMI introduced here is a measure of the amount of information that the interacting genes provide about the trait. This measure is tightly related to the relative genetic variance in particular including all two-SNP/gene interactions (Figure 3). As can be seen and discussed above the ubiquitous SPHK1 and ASAH1 genotypes provided the most information about the sphingolipid network and could be considered as genetic information hubs that express their effects (or information) not exclusively by themself but only through interaction with other genes, i.e., they represent the maximal information and genetic variance accounted for by the entire network. Thus, depending on information contained in a two-SNP interaction the relative impact increases non-linearly to a maximum which is the amount of genetic influence (heritability) on the trait. The hierarchal and non-linear accumulation of relative genetic variance can be understood in same way as two-SNP/gene interactions: two-SNP composite genotypes can be considered as a single entity that will not be functional outside its context i.e., the network or modules in the network, but may provide detectable although small influence on the trait. Any two sets of two-SNP genotypes shares some mutual information about the network i.e., some information is already present, and for this reason either genetic variance or information are cumulative. The argument goes on to the next level and ultimately the information and hence the explained genetic variance asymptotically levels of and reach a maximum, that is the heritability of the trait as depicted in Figure 3.

It was expected that the node degree sequences of the genes were distributed as a power law or Poisson distributed, but to the contrary only a small fractions of the genes were captured by the power law distribution. Most of the genes did not conform to any of several continuos distributions and appears totally random distributed. This trend is confirmed in analysis of effects sizes and variances in which the data sets on average contains more than 5000 samples. However, when only data corrected for multiple testing were included a bipartite structure appeared: the power law distributed data did only capture a fraction of the data, while the Poisson distribution did not fit the entire set of data (corrected for multiple testing); rather, the data set can be split in two components, one captured by the power law, the other by Poisson distributions (as in the famous and widely applied Erdös-Rényi random graph, Erdös and Rényi, 1960). The power law distribution has gathered quite a momentum (for a review see Nacher and Akutsu, 2007), but has also been disputed as a generic description of biological networks (Arita, 2005; Lima-Mendez and van, 2009). More theoretically advanced network modeling is needed (e.g., Dehmer et al., 2014), and as indicated here the prevailing idea that biological networks are scale free may have to be revised.

The influence of the sphingolipid network on the blood pressure regulation was quite large, while the impact of the phosphatidate and redox networks seemed to be limited. Caution should be observed with this conclusion as the selection of genes, mostly from the sphingolipid metabolic network, may have been fortuitous. Other genes or combination of genes could harbor more information than the ubiquitous genotypes above, but the heritability will only increase marginally if at all considering the fits shown in Figure 3. Thus, although possible, it is not likely that any gene-interactions will surpass the ubiquitous genotypes in collated information content. The exception is subpopulation 3, in which the sphingolipid metabolic network only have a minor influence on the blood pressure. The phosphatidate and redox networks could be of importance as only a limited number of SNPs were included, but other networks and pathways are of course possible. The patterns of interactions differs between the subpopulations, but the sphingolipid metabolic network is established to be central to the regulation of the blood pressure.

Only a few of the valid SNPs have previously been associated with any clinical condition (Tables S1a,b). Some of the candidate genes were not included in the analysis as the SNPs turned out to be monomorphic, and for this reason the coverage is not entirely comprehensive (which must be assumed also to be the case for the other networks). In particular, several of the ceramidases, fatty acid binding proteins and S1P- and LPA-receptors were not included. The number of interactions influencing the prevalence of hypertension is rather modest, but may be substantial underestimated as important participants in the networks are missing. Thus, the number of SNPs and genes involved is expected to increase when the inclusion of variations and genes become more comprehensive.

There have been contrasting views about the importance of epistasis in genetic systems. Crow argues that epistasis is unimportant in polygenic directional selection (Crow, 2010) suggesting that additive genetic variance is the driving force. However, there is mounting evidence particular from studies in model organisms that epistasis is essential in governing any traits (Brillinger, 2004; Phillips, 2008; Crow, 2010; Lehner, 2011; Fenger, 2014), not just for intergenic interactions but also for intragenic interactions (Lehner, 2011) as is the case for e.g., ASAH1 and SPHK1. The present studies strongly supports the concept of epistasis as a major source of phenotypic variation. Only a few scattered main effects were detected, but most of genes revealed themself as participants in blood pressure regulation by simple two-SNP/gene interactions. This behavior is a corollary to the weak pairwise correlation in neural networks that impose a strongly correlated state in the entire neural network far beyond what can be explained by the independent state of single neurons (Schneidman et al., 2006; Shlens et al., 2006; Tang et al., 2008). A maximal entropy model was used without any assumptions the mechanistic origin and it were shown that larger networks are completely dominated by correlation effects between neurons. Here, the pairwise genetic interactions completely determined the genetic effects of the networks without any knowledge of the topology necessary.

Generally, the physiological and biochemical interpretation of the interactions revealed in the present study are highly theoretical as both metabolic and functional interactions are possible. Most probably, many of the interactions, apart from the flux of metabolites in the networks, may include elements of stereogenic interactions of the components generating integrated complexes of enzymes (Lehner, 2011) located to the same compartment of the cell e.g., in and at the endoplasmatic reticulum and Golgi apparatus. Basic biochemical and cellular research will be needed to solve these issues.

### Conflict of interest statement

The authors declare that the research was conducted in the absence of any commercial or financial relationships that could be construed as a potential conflict of interest.
